# Genomic Structure of the Luciferase Gene from the Bioluminescent Beetle, *Nyctophila* cf. *Caucasica*

**DOI:** 10.1673/031.006.3701

**Published:** 2006-10-27

**Authors:** John C. Day, Mohammad J. Chaichi, Iraj Najafil, Andrew S. Whiteley

**Affiliations:** ^1^ CEH-Oxford, Mansfield Road, Oxford, Oxfordshire, OX1 3SR, UK; ^2^ Department of Chemistry, Mazandaran University, Babolsar, Iran.; ^3^ Biological Control Centre, Amol, Iran

**Keywords:** Coleoptera, Lampyridae, phytogeny, promoter, *Lampyris turkistanicus*, *Lampyris noctiluca*

## Abstract

The gene coding for beetle luciferase, the enzyme responsible for bioluminescence in over two thousand coleopteran species has, to date, only been characterized from one Palearctic species of Lampyridae. Here we report the characterization of the luciferase gene from a female beetle of an Iranian lampyrid species, *Nyctophila* cf. *caucasica* (Coleoptera:Lampyridae). The luciferase gene was composed of seven exons, coding for 547 amino acids, separated by six introns spanning 1976 bp of genomic DNA. The deduced amino acid sequences of the luciferase gene of *N. caucasica* showed 98.9% homology to that of the Palearctic species *Lampyris noctiluca.* Analysis of the 810 bp upstream region of the luciferase gene revealed three TATA boxes and several other consensus transcriptional factor recognition sequences presenting evidence for a putative core promoter region conserved in Lampyrinae from -190 through to -155 upstream of the luciferase start codon. Along with the core promoter region the luciferase gene was compared with orthologous sequences from other lampyrid species and found to have greatest identity to *Lampyris turkistanicus* and *Lampyris noctiluca.* The significant sequence identity to the former is discussed in relation to taxonomic issues of Iranian lampyrids.

## Introduction

Bioluminescence is a process by which living organisms convert chemical energy into light. Although evident in land animals, bioluminescent organisms predominate in marine environments with only a few groups of terrestrial animals exhibiting the necessary components to generate visible light. These elements are an enzyme, luciferase, and a substrate, luciferin, which are structurally diverse in nature. The majority of bioluminescent beetle species belong to the family Lampyridae of which the firefly species *Photinus pyralis* is the most studied. Firefly luciferase (EC 1.13.12.7) from *P. pyralis* is a 62-kDa enzyme that catalyses emission of yellow-green light (λmax = 560 nm) upon reaction of D-luciferin, ATP and molecular oxygen (White 1971; McElroy and DeLuca 1983; Baldwin 1996; Conti et al 1996; Wood 1995). The cDNA for the *P. pyralis* luciferase was first characterized in 1985 (de Wet et al 1985) and over the years has been extensively studied, including the resolution of its tertiary structure (Conti et al 1996). This wealth of information has been facilitated by the use of luciferase in a range of applications exploiting the bioluminescent function of this enzyme and the requirement of ATP within the reaction. However, work has centred upon a few luciferase sequences obtained primarily from Nearctic and Oriental species. An expansion of luciferase studies to include Palearctic species would present a more complete dataset for phylogenetic studies as well as providing novel sequences for expression purposes. To date only one luciferase sequence from a Palearctic species has been characterized, the European glow-worm *Lampyris noctiluca* (Sala-Newby et al 1996). *Lampyris noctiluca* belongs to the tribe Lampyrini, which is composed of five genera, *Diaphanes, Lampyris, Nyctophila, Pelania* and *Pyrocoelia.* The genus *Nyctophila* was established by Olivier (Olivier, 1884), with the type specimen *N. reichii* Du Val being described in 1859, and is comprised of about 30 known species, most of which are described from Europe and the Middle East (Geisthardt and Satô, in press).

In this study we identify and examine a novel luciferase gene from *Nyctophila* cf. *caucasica* collected from the Amol forest, northern Iran and compare the luciferase gene sequence and promoter region with that of other Lampyridae species.

## Materials and Methods

### Specimens, taxonomy and DNA extraction

*N. caucasica* male and female adult specimens were provided from a maintained colony at Mazandaran University, originally collected from Amol forest, Mazandaran Province, Northern Iran (36°28′N, 52°21′E) and shipped in alcohol to Oxford, England. Total genomic DNA was extracting from a single female specimen using the High Pure PCR Template Preparation Kit (Roche, www.roche.com) according to the manufacturer's instructions.

### PCR and genome walking of the luciferase gene from *N. caucasica*

**Figure 1.**
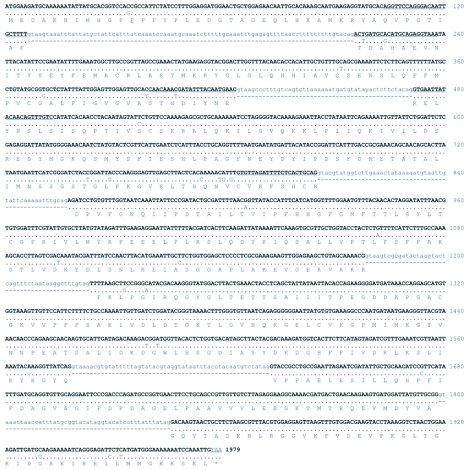
The nucleotide sequence and genomic organization of the luciferase gene from *Nyctophila caucasica* (upper sequence) aligned with the cDNA luciferase sequence obtained from *Lampyris noctiluca,* GenBank accession number X89479 (lower sequence). Sequence identity is illustrated with a dot and deletions are indicated with a dash. Exon sequences are shown in bold uppercase, introns in lowercase. Amino acid differences between *N. caucasica* and *L. noctiluca* are shown underlined.

Based upon the luciferase sequence from *L. noctiluca* (GenBank accession # X89479) two PCR primers, noctlucF1 and noctlucR2 (Table 1) were used to amplify the first 1kb of the luciferase gene from *N. caucasica* (Figure 1). PCR was carried out under the following conditions: initial denaturation at 94 °C for 2 min, ten cycles at 94 °C for 15 sec, 60 °C for 30 sec, and 72 °C for 6 min, twenty cycles at 94 °C for 15 sec, 60 °C for 30 sec, and 72 °C for 6 min plus an additional 5 sec per cycle and a final extension at 72 °C for 7 min. PCR products were ligated into the pGEM®-T Easy Vector System (Promega, www.promega.com/) and ligation mixtures transformed into competent cells of *Escherichia coli* DH5α. Complete nucleotide sequences of PCR products were determined using a dye termination kit and an automatic sequencer (Beckman Coulter, www.beckman.com). From the sequence four primers, Luc5′GW1, Luc5′GW2, Luc3′GW1 and Luc3′GW2 (Table 1) were designed to amplify the remaining downstream sequence of luciferase from *N*. *caucasica* using the Universal GenomeWalker™ protocol (Clontech, www.clontech.com). PCR products were cloned and sequenced as described above. Two primers, NycLuc F1 and NycLuc R1 (Table 1) were used to amplify the entire gene and confirm continuity of sequence.

**Table 1.**
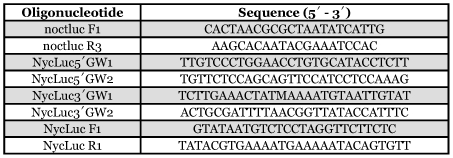
PCR primers used in the amplification of luciferase from *Nyctophila caucasica*.

### Sequence and phylogenetic analysis

DNA sequences from *N. caucasica* were edited and aligned using Sequencher 4.0.5 (Gene Codes Corporation, www.genecodes.com). A consensus sequence was aligned with the cDNA sequence from *L. noctiluca* (GenBank accession # X89479) in order to determine exon/intron positions. The exon positions were further confirmed by comparisons with the *L. noctiluca* gene sequence (accession # AY447204) (Li et al 2003b). All sequences used in the phylogenetic analysis were obtained from the DDBJ/GenBank/EMBL database and accession numbers are as follows: *Cratomorphus distinctus* (AY633557) *Lampyris turkistanicus* (AY742225); Hotaria *papanensis* (AF486802); *Hotaria parvula* (L39929); *Hotaria tsushiman* (AF486801); *Hotaria umnunsana* (AF420006); Lampyris *noctiluca* (X89479); *Luciola cruciata* (P13129); *Luciola lateralis* (U51019); *Luciola mingrelica* (S61961); *Photinus pyralis* (M15077); *Photuris pennsylvanica*( U31240); *Pyrocoelia miyako* (L39928); *Pyrocoelia rufa* (AF328553); *Phrixothrix hirtus* (AF139645); *Phrixothrix vivianii* (AF139044) and *Pyrophorus plagiophthalmus* (S29355). Alignments were carried out using ClustalW followed by manual modification and phylogenetic analysis was carried out using PAUP Vers 4.ob8 (Swofford 2001). The tree was rooted with CG6178 a sequence regarded as a non-bioluminescent ortholog of beetle luciferase from the *Drosophila* genome (Ohba et al 2004).

## Results and Discussion

**Figure 2.**
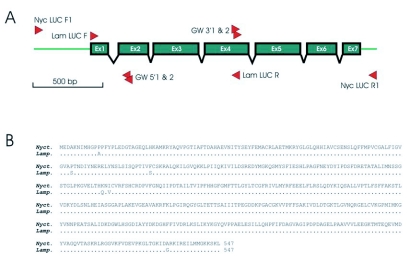
Luciferase gene characterization from *Nyctophila caucasica*. A. Genome organization of the luciferase gene from *N. caucasica* and PCR primer positions. B. Deduced luciferase amino acid sequence alignment of *N. caucasica* (*Nyct*.) and *Lampyris noctiluca* (Lamp.) (Sala-Newby *et al* 1996).

PCR amplification strategies and DNA sequencing were successfully used to isolate and characterize the luciferase gene from a single female *N. caucasica. Lampyris noctiluca* PCR primers LnocF1 and Lnoc R2 were sufficiently conserved to generate a PCR product from *N. caucasica* 1008 bp in size. Sequencing provided information for genome walking primers that amplified products upstream and downstream of the luciferase gene in *N. caucasica.* Primers designed at the ends of the genome walking products were used to amplify the entire luciferase gene and both upstream and downstream regions of the gene as one continuous fragment 3086 bp in length. Sequencing revealed the PCR product to be composed of 810 bp of 5′ sequence upstream of the luciferase start codon and 242 bp of 3′ sequence downstream of the stop codon. From the cloned PCR fragments two alleles were identified denoted as *Ncau1* and *Ncau2*. Four synonymous transitions were identified between the two alleles of which only one was located within an exon. The luciferase gene was composed of seven exons, coding for 547 amino acids, separated by six introns and spanning 1976 bp of genomic DNA (Fig. 1 and Figure 2). The entire 3086 bp sequence, including primer sequence, was deposited in GenBank, accession # DQ072141.

To date, the entire luciferase gene has been characterized from six different Lampyridae species and in all species the luciferase gene is composed of seven exons divided by six introns. The introns are relatively conserved in size, with the most size variation occurring in intron 1, the largest intron present in the *N. caucasica* luciferase gene (Table 2). One of the most interesting differences between the luciferase gene sequences is that found between the two populations of *P. rufa* reported by Li *et. al*. (Li et al 2003a) in which three amino acid substitutions occur with extensive variation between intron sequences from individuals of Chinese and Korean origin (Table 2). This suggests the possibility of cryptic speciation and/or population isolation and highlights the importance of the luciferase gene, especially the intron sequences, as an informative marker for species determination and population genetic studies.

**Table 2.**
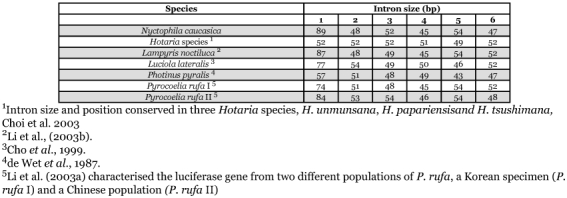
The size of the six introns in the luciferase gene from *Nyctophila caucasica* reported in this paper, compared to those in other lampyrid species.

**Figure 3.**
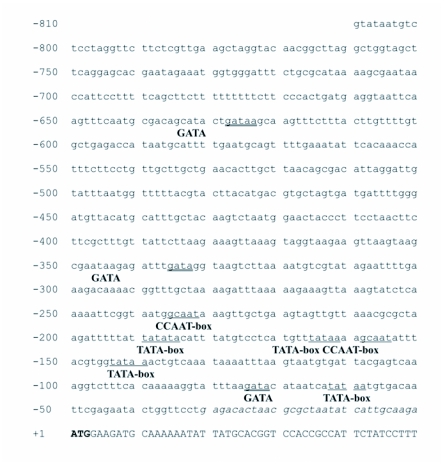
The 5′-flanking nucleotide sequence of the luciferase gene from *Nyctophila caucasica*. Nucleotides are numbered from the translation initiator ATG (bold) with A being position +1. The four putative TATA boxes at positions -63, -144, -166 and -190 and the CCAAT boxes at position -159 and -236 are indicated (underlined). The consensus binding sequences of transcription factor GATA elements are presented double underlined. Also shown is the 5′ end of the *Lampyris noctiluca* cDNA end product indicated by italicised bases (GenBank accession number X89479).

**Figure 4.**
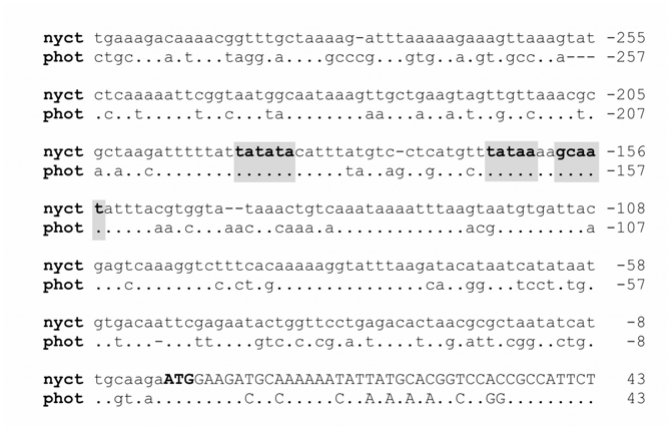
The 5′-flanking nucleotide sequence of the luciferase gene from *Nyctophila caucasica* aligned with orthologous sequence from *Photinus pyralis* (GeneBank accession # M15077). Nucleotides are numbered from the translation initiator ATG (bold) with A being position +1. Two putative TATA boxes and a CCAAT box conserved in both sequences are highlighted in grey.

The 810 bases upstream of the *N. caucasica* luciferase gene were examined for putative promoter sites. Four TATA boxes, two CCAAT boxes and two GATA motifs were identified in the upstream region (Figure 3). Despite extensive sequence differences, comparisons with *P. pyralis* luciferase flanking sequence revealed that three motifs were conserved in both species, two TATA boxes at positions -190 and -166 (positions refer to *N. caucasica* sequence) along with a conserved CCAAT box at position -159 (Figure 4). This suggests the presence of a putative core promoter conserved in Lampyrinae from -190 through to -155. It was not possible to infer a similar core promoter region in members of the Lucolinae due to insufficient identity with *Luciola lateralis* upstream flanking sequence (GeneBank accession numbers U49182 and U51019).

**Figure 5.**
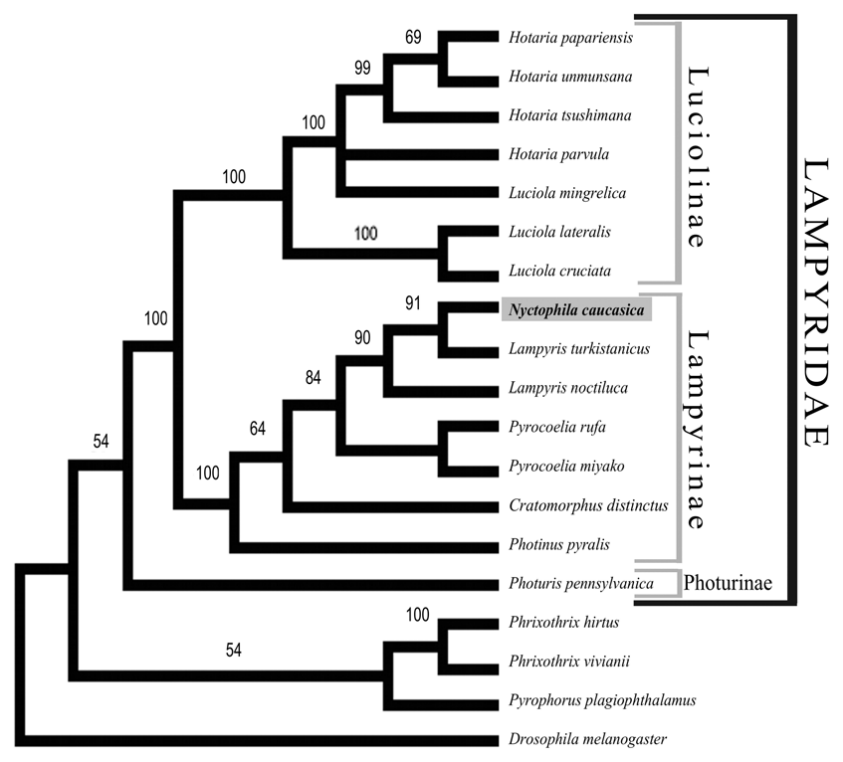
A phylogenetic tree based upon amino acid sequences of the *Nyctophila caucasica* luciferase and fifteen known beetle luciferases. The maximum parsimony tree was obtained by a heuristic search with 1000 bootstrap replicates. Branch numbers refer to bootstrap values.

The deduced amino acid sequences of the luciferase gene of *N. caucasica* showed 98.9% homology to that of *L. noctiluca* (Table 3). Phylogenetic analysis with other bioluminescent beetle luciferases further confirmed that the deduced amino acid sequences of the *N. caucasica* luciferase gene belonged to the subfamily Lampyrinae (Figure 5). Furthermore, with both *Lampyris* and *Nyctophila* along with *Pyrocoelia* species belonging to Lampyrini the luciferase molecular data supports the taxonomic classification of these species down to the tribe level. However, the most identity shared was with *Lampyris turkistanicus,* both at a nucleotide level and an amino acid sequence level, 0.992 and 0.998 respectively, that was greater than the sequence identity with species of its own genus (0.981 and 0.987). The strength of the nucleotide sequence identity, 0.992, suggests a misidentification *of L. turkistanicus.*

**Table 3.**
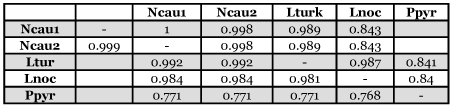
Pairwise identity matrix for the deduced amino acid sequences (above) and CDS (below) among *Nyctophila caucasica* and selected other beetle luciferases. GenBank accession numbers are given in the Materials and Methods.

To date, structural and biochemical studies of beetle luciferin have concentrated on that of *Photinus pyralis.* To our knowledge, luciferin has only been characterized from one other lampyrid, *L. turkistanicus* (Hadj-Mohammadi and Chaichi 1996). Recently the luciferase mRNA (Alipour et al 2004) has been studied providing both enzyme and substrate information comparable to that of *P. pyralis.* However, the lampyrid species that the luciferin was characterized from along with the luciferase mRNA may be in doubt. The high DNA sequence identity of *L. turkistanicus* luciferase to the luciferase gene sequence from *N. caucasica* strongly suggests the possibility of taxonomic confusion. Furthermore, the specimens used in this current study were those obtained from the same forest area that provided specimens for both the luciferase mRNA and luciferin characterisation of *L. turkistanicus.* These data combined with the fact that *L. turkistanicus* has never been reported in Iran (pers. comm. M. Geisthardt) provides strong evidence that *N. caucasica* has, in the past, been misidentified as *L. turkistanicus.* With time we hope to resolve this issue and will eventually provide a bioluminescent system characterized at the luciferin and luciferase level to the same extent as that of *P. pyralis*.
